# Effects of Deposition Temperature and Working Pressure on the Thermal and Nanomechanical Performances of Stoichiometric Cu_3_N: An Adaptable Material for Photovoltaic Applications

**DOI:** 10.3390/nano13222950

**Published:** 2023-11-15

**Authors:** M. I. Rodríguez-Tapiador, A. Jiménez-Suárez, A. Lama, N. Gordillo, J. M. Asensi, G. del Rosario, J. Merino, J. Bertomeu, A. Agarwal, S. Fernández

**Affiliations:** 1 Departamento de Energía, CIEMAT, Av. Complutense 40, 28040 Madrid, Spain; mariaisabel.rodriguez@ciemat.es; 2 Area de Ciencia e Ingeniería de Materiales, Universidad Rey Juan Carlos, Tulipán, s/n, 28933 Móstoles, Spain; alberto.jimenez.suarez@urjc.es; 3 Department of Mechanical and Materials Engineering, Florida International University, Miami, FL 33174, USA; alama043@fiu.edu (A.L.); agarwala@fiu.edu (A.A.); 4 Centro de Microanálisis de Materiales (CMAM), Universidad Autónoma de Madrid, 28049 Madrid, Spain; nuria.gordillo@uam.es; 5 Departamento de Física Aplicada, Universidad Autónoma de Madrid, 28049 Madrid, Spain; 6 Instituto Nicolás Cabrera, Universidad Autónoma de Madrid, 28049 Madrid, Spain; 7 Departament de Física Aplicada, Universitat de Barcelona, 08027 Barcelona, Spain; jmasensi@ub.edu (J.M.A.); jbertomeu@ub.edu (J.B.); 8 Institute of Nanoscience and Nanotechnology (IN2UB), Universitat de Barcelona, 08007 Barcelona, Spain; 9 Centro de Apoyo Tecnológico (CAT), Universidad Rey Juan Carlos, Tulipán, s/n, 28939 Móstoles, Spain; gilberto.delrosario@urjc.es (G.d.R.); jesus.merino@urjc.es (J.M.)

**Keywords:** Cu_3_N thin films, reactive magnetron sputtering, nanoindentation, optical parameters

## Abstract

The pursuit of efficient, profitable, and ecofriendly materials has defined solar cell research from its inception to today. Some materials, such as copper nitride (Cu_3_N), show great promise for promoting sustainable solar technologies. This study employed reactive radio-frequency magnetron sputtering using a pure nitrogen environment to fabricate quality Cu_3_N thin films to evaluate how both temperature and gas working pressure affect their solar absorption capabilities. Several characterization techniques, including X-ray diffraction (XRD), Rutherford backscattering spectrometry (RBS), Raman spectroscopy, scanning electron microscopy (SEM), nanoindentation, and photothermal deflection spectroscopy (PDS), were used to determine the main properties of the thin films. The results indicated that, at room temperature, it is possible to obtain a material that is close to stoichiometric Cu_3_N material (Cu/N ratio ≈ 3) with (100) preferred orientation, which was lost as the substrate temperature increases, demonstrating a clear influence of this parameter on the film structure attributed to nitrogen re-emission at higher temperatures. Raman microscopy confirmed the formation of Cu-N bonds within the 628–637 cm^−1^ range. In addition, the temperature and the working pressure significantly also influence the film hardness and the grain size, affecting the elastic modulus. Finally, the optical properties revealed suitable properties at lower temperatures, including bandgap values, refractive index, and Urbach energy. These findings underscore the potential of Cu_3_N thin films in solar energy due to their advantageous properties and resilience against defects. This research paves the way for future advancements in efficient and sustainable solar technologies.

## 1. Introduction

The beginning of photovoltaic technology dates back to 1839 when physicist Alexander Edmond observed the first photovoltaic effect in silver and platinum electrodes exposed to the sun [1]. In 1873, Willoughby Smith noticed changes in the resistance of selenium bars under light, leading to the first selenium solar cells in 1877. These achieved a modest efficiency of 0.5% [2]. Later, in 1878, Charles Fritts improved efficiency by using selenium between thin layers of metal [3]. Then, in 1905, Albert Einstein proposed the photoelectric theory, which describes the relationship between light and photons [4]. In 1939, Russell Ohl replaced selenium with silicon, discovering the p-type and n-type regions of silicon and the photoelectric effect in p–n junctions. In addition, not long after, in 1940, the first silicon solar cell was developed [5]. However, it was not until the 1970s that photovoltaic technology received renewed interest due to the oil crisis. In 1973, Joseph Lindmeyer and Peter Varadi, driving the development of new materials and techniques, such as amorphous silicon, allowed for more cost-effective solar cell manufacturing and founded Solarex [6,7]. Since then, solar cells have been classified into different generations based on the materials and techniques used in their production. Furthermore, research has been promoted to explore new materials that are more environmentally friendly, affordable, and efficient [8,9,10,11]. The National Renewable Energy Laboratory (NREL) presents how the efficiency of different technologies of solar cells has evolved from 1976 to the present, which signifies the constant efforts made by the scientific and technological community in the pursuit of efficiency improvements [10].

Producing photovoltaic energy using nanostructured semiconductor materials is a very promising field for achieving high efficiency at a reduced cost [12,13,14,15,16]. Among the most notable nanostructures of semiconductors used in the photovoltaic field can be found nanocrystal quantum dots. These structures allow for adjusting the wavelengths of light absorption and emission by adapting their structural properties due to quantum confinement effects [17]. Nanowires aim to collect more light with less material for greater sustainability [18]; in the same way, thin films are also a reliable choice [19]. However, when considering semiconductors containing nitride with metals, such as Indium (In), Tantalum (Ta), and Gallium (Ga), there is a notable drawback to contend with: they fall under the category of critical and strategic raw materials according to the report presented by the European Union (EU) in January 2023 [20]. These materials, either due to the hazardous nature of their extraction or their geographic location, pose significant challenges when it comes to procurement. The importance of these metals is critical for the EU. Without them, the EU’s position as a technologically advanced and economically decarbonized power may be at risk in the near future since these materials are essential for clean-energy sectors. Therefore, there is a need to search for cheaper and more accessible ecofriendly materials. In this sense, copper nitride (Cu_3_N) is considered as a potential alternative choice to replace these expensive and hard-to-obtain materials. In the photovoltaic field, Cu_3_N thin films are particularly interesting due to their favourable environmental characteristics and their composition based on abundant elements found on the planet. Cu_3_N is a metastable semiconductor, which means that, above 300 °C, it decomposes into metallic copper (Cu) and nitrogen (N) [21].

Cu_3_N thin films, with thicknesses less than 100 nm and grain sizes between 11 nm to 100 nm, can be produced [22,23], exhibiting a cubic anti-ReO_3_ crystalline structure with a lattice parameter of 0.3817 nm and presenting a smooth granular morphology [22,23,24]. Their optical properties are basically determined by the band gap, with experimental values ranging from 1.17 to 1.69 eV for the indirect band gap and from 1.72 to 2.38 eV for the direct band gap [25,26]. It also displays a variation in electrical conductivity, which can range from 10^−3^ to 10^2^ S/cm, classifying it as an insulator, semiconductor, or conductor, respectively [21,27]. Furthermore, the material’s composition during the film-deposition process can control the type of doping, determining whether the film is n-type (rich in Cu) or p-type (deficient in Cu) [28,29,30]. A notable characteristic of copper nitride is its defect-tolerant electronic structure. These defects present in the material do not significantly affect its properties, unlike conventional semiconductors, which have their band gap altered by defects. On the contrary, copper nitrides, even in the presence of crystallographic defects, maintain their character of the maximum valence band (VBM); that is, the highest energy at which electrons are found in the valence band before being excited to the conduction band when additional energy, such as light or heat, is provided. This was demonstrated by Andriy Zakutayev et al. [31], who compared the results between GaN and Cu_3_N. Theoretical calculations show Cu_3_N’s antibonding valence band has shallow intrinsic defects and no surface states, unlike GaN. All these parameters that can make Cu_3_N thin films into suitable photovoltaic absorbers can be designed and easily modified when the material is fabricated by using the reactive magnetron-sputtering method. This method is considered as one of the most employed in the fabrication of nanostructures and thin films [32,33]. This is because the growth parameters can be easily controlled, thus managing the thickness, the deposition rate, and the coating uniformity [34]. Moreover, it is highly flexible [35] and enables the control of key parameters, such as substrate temperature, working pressure, and bias voltage, among others [22,24,32,36,37,38,39,40,41,42]. Specifically, radio-frequency (RF) magnetron sputtering offers the advantage of generating fewer impurities compared to chemical methods, thus enhancing the material’s purity and quality. In the study of thin Cu_3_N films fabricated via RF magnetron sputtering, nanoindentation is crucial. This is because it precisely measures the mechanical properties, evaluates the film adhesion, optimizes manufacturing, and ensures quality control for enhanced performance in various applications. Taking this into account, in this work, in pursuit of developing Cu_3_N thin films to be used as potential solar absorbers, a comprehensive analysis of the material’s behaviour is undertaken. This study involves determining the influence of key sputtering parameters, such as substrate temperature and working pressure, using pure nitrogen (N_2_), on the stoichiometry and the relevant properties of the thin films. Our ultimate goal is to assess which deposition conditions are most suitable to ensure that this ecofriendly material shows the greatest solar absorption capacity. 

## 2. Materials and Methods

The film-deposition process was carried out using a single-chamber sputtering system from MVSystem LLC (Golden, CO, USA). The substrates were Corning 1737F glass (Corning Inc., New York, NY, USA) and polished single-crystalline p-type floating zone silicon (c-Si) wafers with <111> orientation (TOPSIL Global Wafers A/S, Frederikssund, Denmark). The initial step involved removing the oxide layers from the silicon wafers and cleaning the Corning glass to remove any potential lab-storage residue. For the silicon wafers, a cleaning procedure was carried out by immersing them in a 1% hydrofluoric acid (HF) solution diluted with deionized water for 5 min. The glass substrates were thoroughly cleaned and immersed in an isopropyl alcohol solution within the ultrasonic equipment, followed by a Milli-Q water rinse step for 3 min. The sputtering target used consisted of high-purity (99.999%) metallic copper (Lesker Company, St. Leonards-on-Sea, UK). The base pressure chamber was maintained at 2.6 × 10^−5^ Pa, whereas the working pressure was adjustable to 5.0 and 3.5 Pa, respectively, by using a butterfly valve. A 50 W of RF (13.56 MHz) power was applied and the distance between the substrate and the target was kept at 10 cm. The sputtering atmosphere consisted of pure nitrogen (N_2_) (99.9999%), and the gas flow was controlled to 20 sccm through MKS Mass Flow Controllers (MFCs) provided by MKS Instruments (Andover, MA, USA). The sputtering process was carried out at room temperature (RT) and substrate temperatures of 100, 150, 200 and 250 °C for 30 min. 

The film thickness was measured with a Dektak 8 profilometer (Bruker, San José, CA, USA). A tip force of 68.67 µN and a scan size of 2000 µm were used in all cases. To measure the film thickness, a step with a well-defined edge was created on the film’s surface. The measurement along the profile was performed up to 10 times, discarding the lowest and highest values before calculating the average value. This provides an accurate measurement of the film’s thickness across its entire surface. To assess the crystallinity of the Cu_3_N films, an X-ray diffraction (XRD) analysis was performed using a commercial system (model PW3040/00 X’Pert MPD/MRD) (Malvern Panalytical Ltd., Malvern, UK) with Cu-Kα radiation (λ = 0.15406 nm). The range of 2θ analysed was from 10° to 60°, with a step interval of 0.01° and a time of 20 s per step. In addition, the effect of different substrate temperatures on the surface morphology was examined using a JEOL JSM 7600F scanning electron microscope (JEOL Ltd., Akishima, Japan), equipped with a field-emission Schottky electron gun, an in-lens secondary electron detector, and a scanning system. Ion-beam analysis techniques (IBA), including Rutherford and non-Rutherford backscattering spectroscopy (RBS and non-RBS), combined with nuclear reaction analysis (NRA), were used to determine the chemical composition of the films. RBS and non-RBS measurements were carried out at the CMAM/UAM [43] using a He^+2^ beam at the energies of 2.0 and 3.7 MeV, respectively. For an accurate assessment of the nitrogen concentration, we have combined the non-RBS measurements, where the relative cross section (σ_n_/σ_R,_ of the ^14^N(α,α)^14^N is approximately 9, with the detection of protons emitted from the nuclear reaction channel, ^14^N(α,p_0_)^17^O, which coincidentally becomes active at 3.7 MeV. Additional information regarding the non-RBS measurement and analysis procedure is available in [44]. Ultimately, the chemical composition of the films was determined by comparing the measured and simulated spectra derived from the RBS and non-RBS data. To perform these simulations, we utilized the commercial computer code SIMNRA [45,46]. The molecular structure was determined using a dispersive spectrometer confocal Raman microscope capable of 3D XYZ confocal Raman imaging. This system was equipped with a 532 nm laser, two diffraction gratings (600 and 1800 gr/nm), three objectives (5×, 75×, and 100×), and the ability to acquire photocurrent mappings (Horiba LabRam soleil, Longjumeau Cedex, France). The nanomechanical measurement of nanoindentation was carried out in the Tribo-Indenter TI 900 equipment from the Hysitron Company (Eden Prairie, MN, USA). The Berkovich tip that was used in the experiment has standard geometry (semiangle of 65.3 degrees and apex half angle of 76.9 degrees) and a tip radius of 100 nm. The loading profile consists of 3 segments: a loading segment of 10 s till the load reaches 300 µN, a holding segment of 2 s at the peak load and an unloading segment of 10 s till the load drops to 0 µN. Finally, to assess the suitability of Cu_3_N as a solar absorber, optical transmittance spectra were acquired at RT and normal incidence using a Perkin Elmer Lambda 1050 UV–VIS–NIR spectrophotometer (PerkinElmer, Waltham, MA, USA). The optical bandgap (*E*_g_) energies for indirect and direct transitions were calculated from these spectra. The optical properties of Cu_3_N thin films deposited on glass were analysed by UV–VIS–NIR optical spectroscopy and photothermal deflection spectroscopy (PDS). Transmittance (*T*_opt_) and reflectance (*R*_opt_) spectra were obtained using a PerkinElmer Lambda 950 UV-Vis spectrometer with a 150 mm integrating sphere.

## 3. Results

The Cu_3_N thin films were deposited on Si and Corning glass substrates at different substrate temperatures, obtaining stable and highly adherent coatings. The details of the films, such as thickness and deposition rate, are shown in Table 1, as a function of the substrate temperature and the working pressure.

According to the information provided in Table 1, the deposition rate decreased with the substrate temperature. This phenomenon is attributed to the metastable nature of copper nitride, which can lead to a nitrogen re-emission effect on the thin film, thereby interfering with its structure and adversely affecting film growth. A turning point is observed at 100 °C, where a decrease in thickness is evident as the temperature increases. These findings will be further elucidated through the utilization of various characterization techniques in our study.

### 3.1. Structural Properties

#### 3.1.1. X-ray Diffraction Analysis

**Figure 1:**
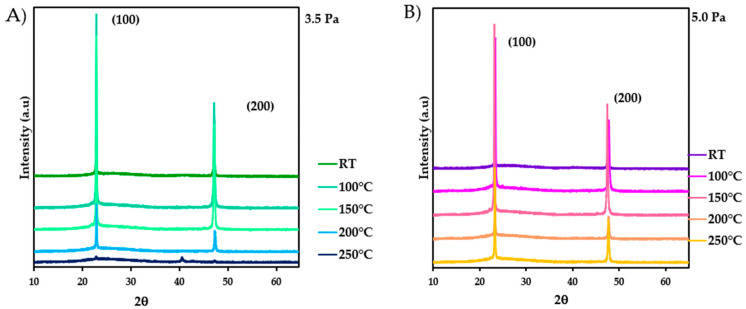
X-ray diffraction patterns of Cu_3_N thin films deposited on glass at different temperatures for the two working pressures of (**A**) 3.5 Pa and (**B**) 5.0 Pa.

Figure 1 illustrates the X-ray diffraction patterns of the Cu_3_N thin films deposited on glass at different substrate temperatures and working pressures. From the XRD analysis, the polycrystalline nature of the films was determined, exhibiting an anti-ReO_3_ crystal structure (card number 00.047.1088). Two peaks were well identified around 2θ~23.28°, corresponding to the (100) crystallographic plane, and 2θ~47.60°, corresponding to the (200) one. An exception in the preferred orientation can be observed in the XRD pattern of the sample deposited at 3.5 Pa and 250 °C. Here, a dominant peak emerged at 2θ~40.90°, related to the (111) diffraction plane. In addition, a very weak (100) diffraction peak was embedded in an amorphous matrix. The intensity of the peaks was observed to increase as the temperature rose, reaching a maximum value at 150 °C. On the other hand, in the case of the Cu_3_N thin film prepared at 250 °C and 5.0 Pa, the crystalline structure persisted, unlike that deposited at the lower pressure of 3.5 Pa. This fact could be attributed to the film’s higher nitrogen atom density, which would facilitate the increase of the Cu-N bonds through the chemical reactions with the copper atoms released during the sputtering process.

Through Bragg’s law (Equation (1)), it was possible to determine the lattice constant (*a*), which, in the literature, the lattice constant is 0.3815 nm [47], where *d* represents the interplanar distance, and *h*, *k*, and *l* are the Miller indices [48].

(1) $$d\left(hkl\right)=\frac{a}{\sqrt{\left(h^{2}+k^{2}+l^{2}\right)}}$$

To calculate the grain size (*τ*) (Equation (2)), the Debye–Scherrer equation was employed, where *K* is a constant with a value of 0.9, λ is the wavelength of X-rays (0.15406 nm), *θ* is the diffraction angle, and *β* is the full width at half maximum (FWHM) of the preferred orientation [49].

(2) $$\tau =\frac{K\lambda }{\beta \cdot \cos\theta }$$

In the formation of Cu_3_N, imperfect crystals are common, and these imperfections significantly affect the material’s properties. When applying a thin film to a substrate, it causes substrate bending due to stress. In crystalline materials undergoing elastic deformation, the lattice spacing directly relates to the deformation magnitude. In nanocrystalline materials, defects, like grain size, vacancies, internal stress, and dislocations, create excess free volume at the grain boundaries. This free volume, along with surface and interfacial tensions, adds to overall material stresses. Thus, microstrain (*ε*), dislocation densities (*δ*) (lines/nm^2^), and the number of crystals per unit area (*N_c_*) (nm^−2^) were also determined from the Scherrer Equations (3)–(5) [50]:

(3) $$\varepsilon =\frac{\beta \cdot \cot\theta }{4}$$

(4) $$\delta =\frac{1}{\tau }$$

(5) $$N_{c}=\frac{d}{\tau^{3}}$$

Table 2 summarises the values calculated from XRD patterns using the Scherrer and Debye–Scherrer equations. The FWHM decreased as the substrate temperature increased. Additionally, an inverse relationship between the lattice parameter and grain size was observed as the substrate temperature increased. This can be due to the additional precipitation of nitrogen around the crystallites, suppressing their growth and affecting the dimensions of the crystal grains. The increase of the substrate temperature from room temperature to 250 °C may lead to a reduction in the nitrogen concentration within the formed Cu_3_N crystals, as will be deduced from the chemical composition shown later. This decrease in nitrogen concentration is explained by the migration of unbound nitrogen atoms from vacant interstitial sites within the structure to the crystallites themselves [48].

Furthermore, the microstrain led to a broadening of the XRD lines without affecting the main peak position. These broadenings were caused by nonuniform displacements of atoms concerning their reference positions in the lattice, and the microstrain within the domains was considered lattice defects [51]. The calculated values indicated that the microstrain decreased when the grain size increased. This result could be due to the reduction in the volume occupied by atoms arranged within the crystal cluster as the substrate temperature rises. Additionally, the density of dislocations, defined as the length of dislocation lines per unit volume, can be associated with a crystal imperfection related to a misalignment of the lattice. In Table 2, it is observed that the density of dislocations decreased as the substrate temperature increased, just like the number of crystallites per unit volume, which depends on the crystal formed in the Cu_3_N thin film. In other words, as the substrate temperature increased, there was a decrease in the number of crystallites per unit volume, as well as a decrease in the density of dislocations, the microstrain, and the lattice parameter, while the grain size increased. Additionally, it is observed that the predominant crystal plane is (100), and this preferred orientation changed as the temperature increased. All of these phenomena could be explained by the release of nitrogen as the temperature increased.

#### 3.1.2. RBS and Raman 

**Figure 2:**
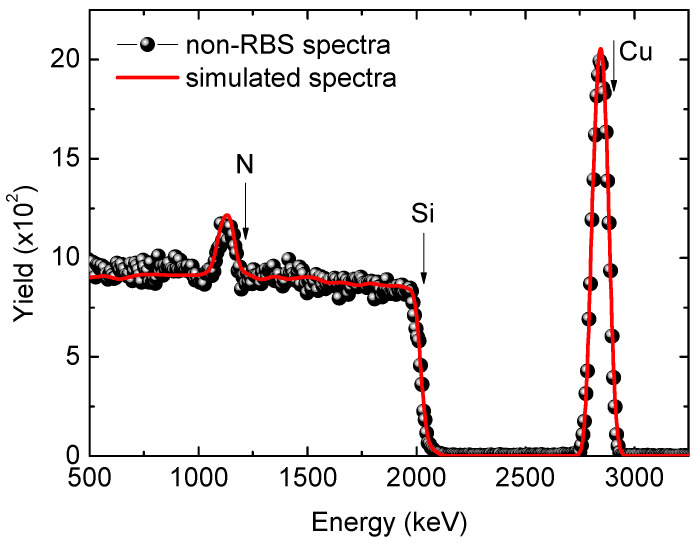
Typical non-RBS spectrum (in black) and simulated spectrum (in red) of a Cu_3_N sample deposited at 3.5 Pa at room temperature.

Figure 2 presents both typical measured and simulated non-RBS spectra. For the analysis of the RBS spectra, the SIMNRA simulation program version 7.03 (https://mam.home.ipp.mpg.de/ (accessed on 8 July 2023)) has been used. This is a Microsoft Windows program for simulating the energy spectra of charged particles, allowing the analysis of ion beams with energies ranging from keV to MeV. The simulation methodology is as follows. All known experimental data are input into the program, including energy, fluence, charge, current, geometry, the elements composing the target material to be analysed, and the ion-atom cross sections within the energy range used (the cross sections are included in the software database). The sample thickness and roughness are other known parameters, as it has been measured using complementary techniques like profilometry and AFM; however, it is also an adjustable parameter. Once all the data are entered, the only variable left is the elemental composition. The goodness of fit between the simulated and experimental spectrum is determined through the “chi-squared” value. These spectra provide clear evidence that the sputtered films consisted of a thin Cu_3_N layer on a Si substrate. The layer exhibited no discernible impurity traces, except for the presence of oxygen (O) and carbon (C), which can be typically introduced during the sputtering process conducted at elevated temperatures. Table 3 displays the atomic concentrations of nitrogen and copper in the Cu_3_N films, as determined through the data acquired from RBS, non-RBS, and NRA techniques.

Building upon the attained results concerning the percentage concentrations of nitrogen (N %) and copper (Cu %) through IBA, we have undertaken fundamental calculations. These calculations have facilitated a comprehensive assessment of the stoichiometric equilibrium state within the Cu_3_N compound. It is clear that, at low substrate temperatures, the Cu/N ratio remained approximately at three, indicating a constant proportion between these elements. However, at temperatures above 150 °C, more scattered values in the concentration of these elements were observed. This phenomenon could be explained by the re-emission of nitrogen, as also evidenced in the X-ray diffraction (XRD) results. This behaviour is attributed to the metastable nature that the compound Cu_3_N acquires as the temperature increases.

**Figure 3:**
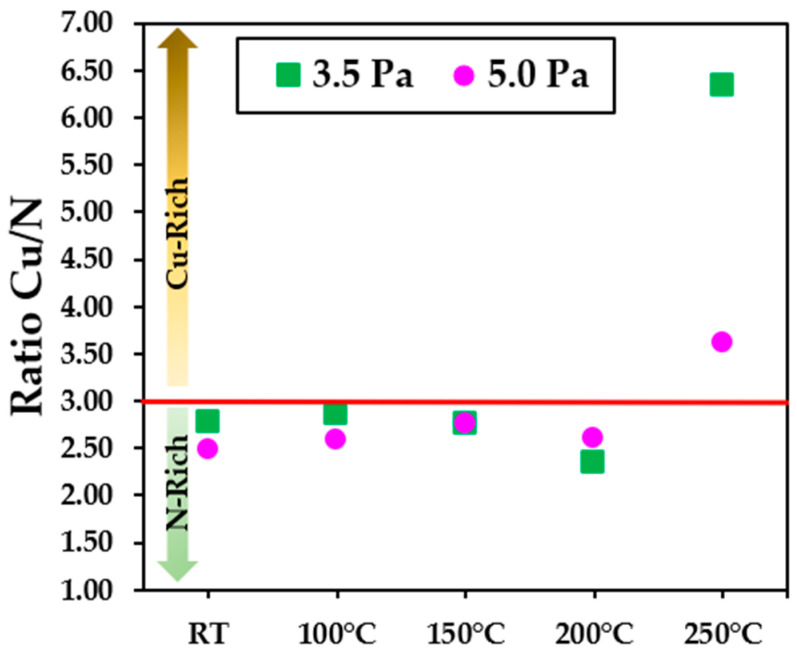
Ratio of Cu/N determined by RBS as a function of the substrate temperature.

Figure 3 provides a graphical representation of this phenomenon, allowing for a clearer visualization of how the concentration of nitrogen atoms fluctuates with respect to the substrate temperature. Consequently, it could be assumed that maintaining a low substrate temperature and a working pressure of 5.0 Pa could be an effective strategy to suppress the formation of undesired metallic phases in the thin film structure of Cu_3_N. This has important implications, as it could facilitate the reproducibility of samples with specific compositions required for its use as a solar absorber or in other applications.

**Figure 4:**
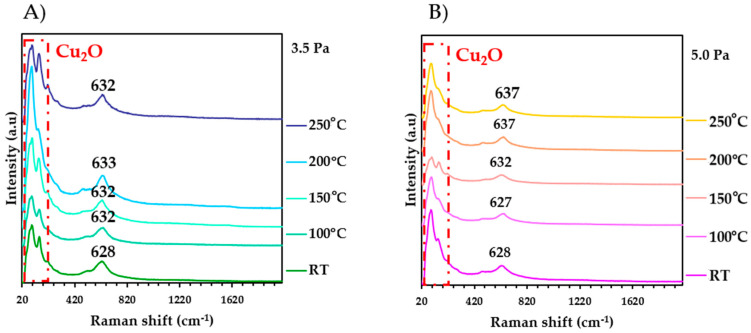
Raman spectra for Cu_3_N films at different temperatures. (**A**) 3.5 Pa and (**B**) 5.0 Pa.

In addition to the analyses using RBS, Raman spectroscopy was carried out to investigate the formation of Cu-N bonds as a function of the substrate temperature and their response to the deformations in the material deposited on a silicon substrate. The theoretical value of the Raman shift for the Cu_3_N thin films is approximately 634 cm^−1^. As can be seen in Figure 4, as the temperature increased, there was a slight shift of Raman peaks towards higher wavenumbers; this effect was most pronounced at the working pressure of 5.0 Pa. In addition, several peaks were observed around 94 cm^−1^, characteristic of Cu_2_O. It is worth noting that no copper (I) oxide peaks were detected in the previous XRD patterns, indicating that the temperature-induced changes may be primarily associated with both surface reactions and the formation of a surface oxide film, rather than extensive bulk oxidation. A more detailed analysis is required to have a full understanding of the specific oxidation mechanisms and their impact on the material’s properties. 

The observed Raman shifts can be attributed to the structure of the material and its response to stresses generated within the film as the temperature increases. This phenomenon is correlated with the relationship between the Raman peaks and the microstrain obtained from XRD analysis, as shown in Table 4. It is evident that the Raman peaks tend to shift towards higher wavenumbers as the temperature increases simultaneously with a reduction of the microdeformations. This behaviour could be related to the re-emission of nitrogen as the substrate temperature increased, suggesting that the films fabricated at a working pressure of 5.0 Pa experienced less deformation compared to the films fabricated at 3.5 Pa. Furthermore, through RBS, it was observed that the films deposited at 5.0 Pa of working pressure had a higher atomic percentage of nitrogen.

These RBS and Raman results provide a deeper understanding of how Cu_3_N thin films respond to the different substrate temperatures and working pressures, which could have important implications for their application in specific technologies and their long-term stability.

#### 3.1.3. Scanning Electron Microscopy Analysis

**Figure 5:**
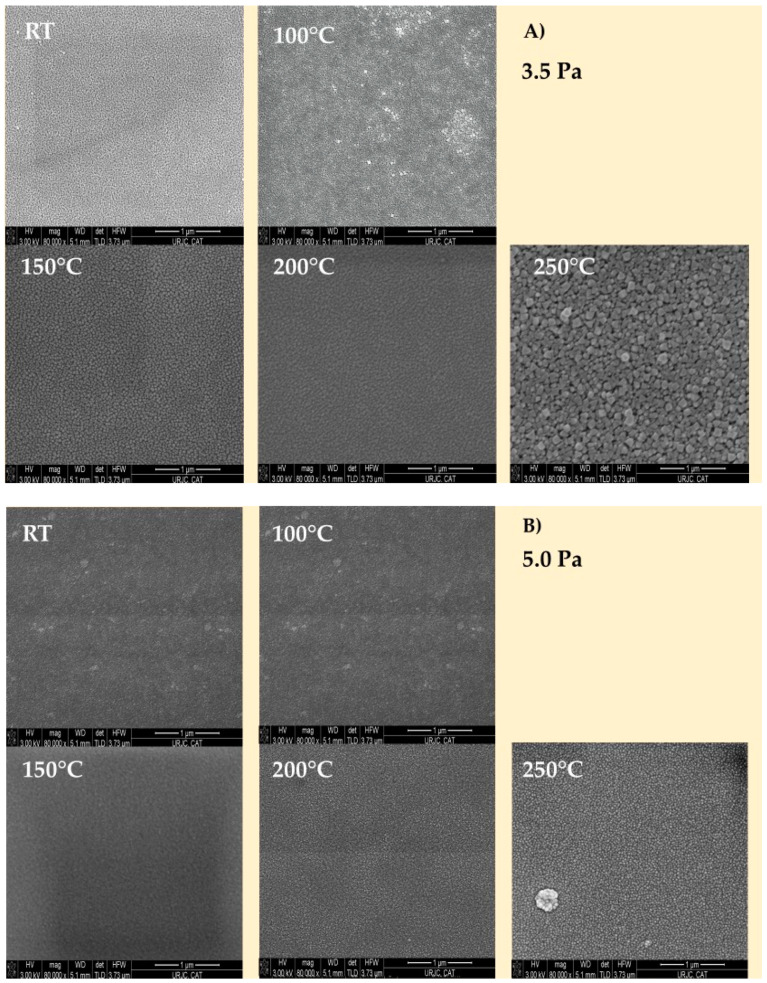
FE-SEM images of Cu_3_N films fabricated at different substrate temperatures and working pressures, deposited on silicon substrates of (**A**) 3.5 Pa and (**B**) 5.0 Pa.

Figure 5 depicts the surface morphology of Cu_3_N thin films deposited at different substrate temperatures and working pressures, characterized by SEM. The images reveal a flat surface morphology, uniformly composed of a granular structure. Additionally, in our recent work, we reported that these films exhibit a columnar structure [42]. This finding reinforces what was previously mentioned in the XRD analyses. As the temperature increased, a noticeable increase in grain size was observed. In other words, these crystallites tended to merge or aggregate, and, hence, this dynamic appears to be associated with the re-emission of nitrogen that occurs as the temperature rises.

**Figure 6:**
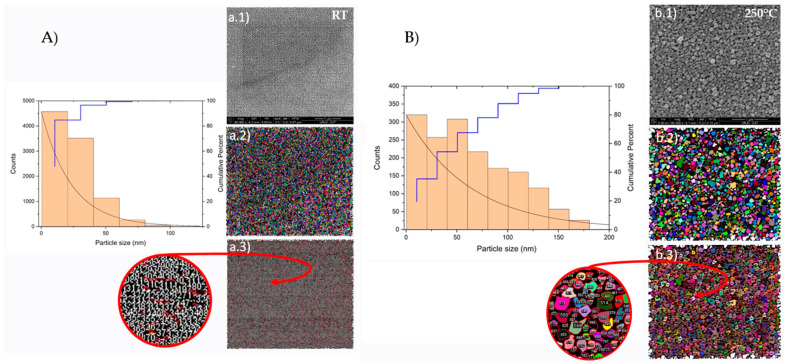
Grain-size determination for thin films prepared at different temperatures: (**A**) RT and (**B**) 250 °C. Fe-SEM images (**a.1**,**b.1**); Segmentation of the input images (**a.2**,**b.2**); and quantification of the segmentation of the input images (**a.3**,**b.3**).

Figure 6 illustrates the grain-size determination from the SEM images for the samples deposited at RT and 250 °C. The thin film prepared at 3.5 Pa and RT showed an average grain size of 23.3 ± 16.2 nm, whereas, for the sample deposited at 3.5 Pa and 250 °C, that value was 62.9 ± 42.5 nm. In comparison with the same parameter extracted from XRD (see Table 2), these values were a little far, although it is worth noting that if the standard deviations are considered, they were quite similar. This observed discrepancy can be associated with the size of the surface area analysed with XRD, typically an irradiated area of 10 mm × 5 mm (≈50 mm^2^), quite superior to the analysed by SEM (≈20 μm^2^). In spite of this, the increasing tendency of the grain size with the substrate temperature is regardless of the characterization technique. 

### 3.2. Nanomechanical Properties

#### Nanoindentation

Table 5 summarises the elastic modulus and the hardness obtained for the Cu_3_N thin films deposited on Corning glass as a function of the substrate temperatures and the working pressures determined by nanoindentation tests. It can be clearly seen that the hardness decreases as the substrate temperature increases, regardless of the working pressure value. This phenomenon could be attributed to the relaxation of residual stress at elevated temperatures. As the temperature rises, the lattice structure of the film tends to adjust, leading to a reduction in the hardness of the material. This is a common behaviour observed in thin films subjected to high temperatures [52]. In addition, the XRD results provided further insights into the changes observed in the mechanical properties of the films. There was a noticeable increase in the crystal size, as detected by XRD. This increase in crystal size can be directly linked to the decrease observed in the hardness during the nanoindentation tests. Larger crystal sizes typically lead to a reduction in the material’s hardness, as there is a more extensive atomic lattice for the dislocation movement within the material.

Interestingly, the elastic modulus exhibited a complex behaviour. Initially, it increased, suggesting a strengthening effect as the temperature initially rose. However, this trend reversed as the temperature continued to increase. The most significant decrease was observed at 150 °C and 200 °C for films deposited at 3.5 Pa and 5.0 Pa, respectively. This decrease in the elastic modulus aligns with the substantial changes observed in the Cu/N ratio. The presence of other phases, as mentioned earlier, could have a direct impact on the mechanical properties of the coating, influencing its elastic modulus. In summary, the mechanical properties of the films were highly influenced by the temperature and the working pressure during the film deposition. The decrease in hardness could be attributed to stress relaxation, while the increase in crystal size and the subsequent decrease in hardness were in line with the changes obtained in the elastic modulus [52].

### 3.3. Optical Properties

**Figure 7:**
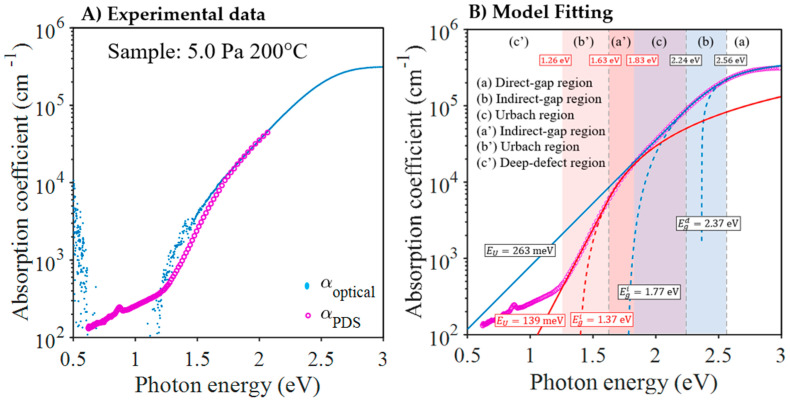
Absorption coefficient *α* versus photon energy (*E*) derived from optical and PDS measurements of the sample deposited at 5 Pa and 200 °C. (**A**) Experimental data—the blue dots are the absorption coefficients α_optical_ derived from the UV–Vis–IR spectroscopy measurements and the pink circles are the absorption coefficients α_PDS_ derived from the PDS measurement. (**B**) Fitting of *α*(*E*) with the Urbach-Tauc/indirect–Tauc/direct model. The blue curve represents the usual fit of the strong absorption front and the red curve is the fit of a second front that appears exceptionally in the weak absorption zone of this sample.

The optical properties of the films deposited on Corning glass were determined by optical (UV–Vis–NIR) spectroscopy and photothermal deflection spectroscopy (PDS). Optical spectroscopy makes it possible to accurately determine the absorbance of a sample in the strong absorption region of the spectrum by measuring its transmittance and reflectance. It is the most widely used technique for determining the optical band gap of a material. However, because it is an indirect measure, its sensitivity is limited, and it generally does not allow reliable measurements of absorbances below 1%. In contrast, PDS is a quantitative and direct method based on the detection of temperature changes caused by absorption. In this model, the Cu_3_N absorption edge is described by a double indirect–direct bandgap and the Urbach tail. Figure 7B shows the final fit of the experimental data of Figure 7A. PDS can accurately measure absorptions lower than 1%, which enables the detection of weak subgap absorption arising from semiconductor defects. PDS also offers a reliable measurement of the exponential decay of the absorption front, facilitating the extraction of Urbach energy (E_U_), which characterizes the structural disorder of the semiconductor.

In this work, we combine optical and PDS measurements to obtain the absorption coefficient spectrum across a broad range of energies. An illustration of this spectrum can be found in Figure 7A. From the full spectrum, we determine the most significant optical parameters of Cu_3_N, including band gaps and Urbach energy, by utilizing the model fit described in reference [41].

**Figure 8:**
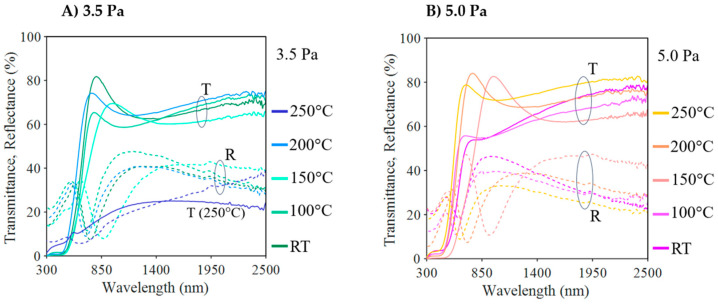
Optical transmission and reflection spectra of the Cu_3_N films deposited at different temperatures and the working pressures of (**A**) 3.5 Pa and (**B**) 5.0 Pa.

Figure 8 displays the transmittance and the optical reflectance spectra for the Cu_3_N samples deposited at different temperatures at the two working pressures in the study. Optical spectra always showed a strong absorption zone in the visible wavelength range and a transparent zone (with an absorption of less than 1%) in the infrared wavelength range. The only anomalous behaviour was observed in the sample with the highest amount of copper; where a layer was deposited at 250 °C and 3.5 Pa, which exhibited strong absorption throughout the entire spectrum.

In the transparent region, there was a minimum of transmittance (corresponding to a maximum of reflectance), which can be explained by the coherent interference of light due to the multiple reflections. The location and value of this minimum depend on the thickness of the layer and its refractive index. Assuming that the layer is homogenous and perfectly smooth and that the refractive index, *n*_∞_, remains constant in the transparent region, the transfer-matrix method (TMM) can be utilized to fit the experimental data, resulting in the determination of *n*_∞_ and the thickness of the layer.

**Figure 9:**
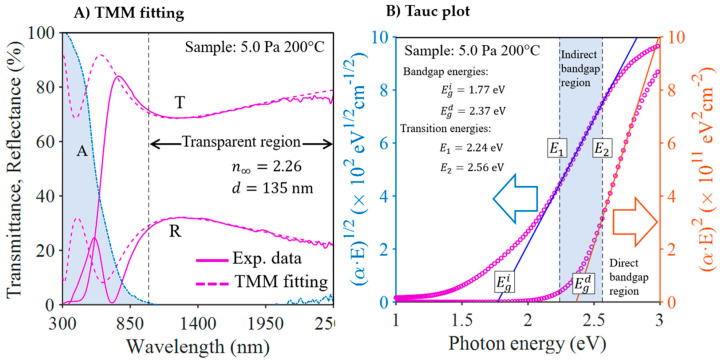
Processing of the optical data of the sample deposited at 5.0 Pa and 200 °C. (**A**) TMM fitting of transmittance and reflectance to obtain the thickness and the refractive index *n*_∞_ of the Cu_3_N thin film. The shaded area represents the optical absorbance (1 − *T* − *R*). (**B**) Plot of (*αE*)^1/2^ versus photon energy *E* for the determination of the indirect band gap *E*_g_^i^, and the plot of (*αE*)^2^ versus *E* for the determination of the direct band gap *E*_g_^d^.

Figure 9 pictures the case of the sample deposited at 5 Pa and 200 °C. Note that the thickness is slightly different from that obtained with the profilometer (see data in Table 1), which can be explained by a lack of homogeneity of the material deposited by RF magnetron sputtering due to the sputtering geometry. Finally, it was found that the thicknesses obtained by the optical fitting could differ by up to 10% from those obtained with the profilometer.

Table 6 displays the extracted refractive index values (*n*_∞_) from the fits. The overall tendency indicates a gradual decrease in *n*_∞_ with increasing deposition temperature. From the values of *n*_∞_ and optical thickness, the absorption coefficient *α* in the “transparent” zone can be determined by comparing the experimental PDS absorption to that obtained through TMM calculation. In the region of strong absorption, the refractive index showed a significant dependence on the wavelength [41], and the TMM calculation with constant *n*_∞_ is not feasible. However, internal reflections in this case are negligible and it is possible to calculate the absorption coefficient directly from the internal absorbance, *A*_opt_/(1 − *R*_opt_), and the thickness. A detailed procedure description can be found in [41].

**Figure 10:**
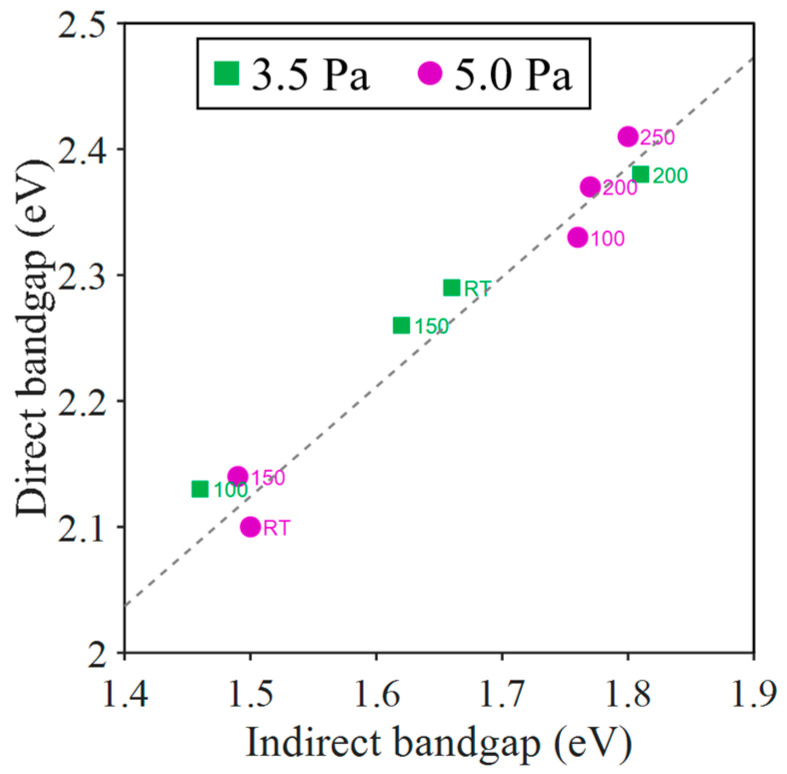
Direct bandgap value *E*_g_^d^ obtained by fitting *α*(*E*) as a function of the corresponding indirect bandgap value *E*_g_^i^. Green squares represent the samples deposited at 3.5 Pa and pink circles; the samples deposited at 5.0 Pa. The deposition temperature is included as the number next to the symbol. The dashed line is a guide for the eye.

**Figure 11:**
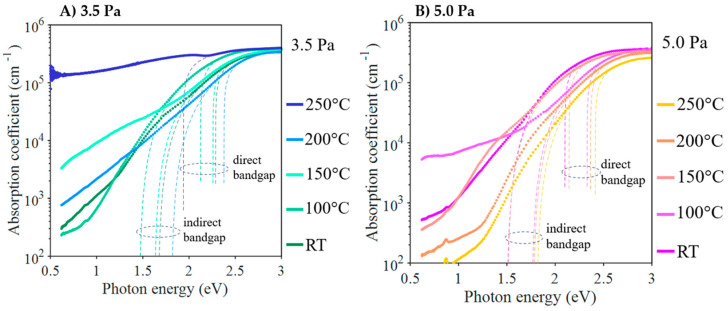
Absorption coefficient spectra of Cu_3_N layers deposited at different temperatures and the working pressures of (**A**) 3.5 Pa and (**B**) 5.0 Pa.

As mentioned above, the Tauc analysis of the absorption coefficient of Cu_3_N, as deduced from the optical measurements, indicates the existence of a double indirect–direct bandgap. As an example, Figure 9B displays the double Tauc plot of the sample deposited at 5 Pa and 200 °C. If the photon energy exceeds a certain “transition” energy level of *E*_2_ (2.56 eV in this example), the absorption is caused by the direct gap, and the Tauc fit allows for the determination of the direct band gap *E*_g_^d^ (2.37 eV in this example). For lower photon energies, the absorption can be explained by the indirect gap model. As a result of the Tauc fit, the indirect band gap *E*_g_^i^ gap is obtained (1.77 eV in this example). It is important to note that the transition from the direct to the indirect band-gap regime occurs smoothly with continuous variations of both *α*(*E*) and its derivative d*α*/dE for the transition energy, *E*_2_. Finally, Table 6 shows the values for both the direct and indirect band gaps determined by fitting the absorption coefficients. As shown in Figure 10, there is a clear correlation between the values of the two gaps; higher *E*_g_^d^ values always correspond to higher *E*_g_^i^ values. Figure 11 pictures the experimental plots of the absorption coefficient for all the samples with the corresponding fits, according to the indirect and direct Tauc models. In conclusion, the samples deposited at a higher temperature (>200 °C) exhibited the highest band gaps (*E*_g_^i^ > 1.7 eV and *E_g_*^d^ > 2.3 eV). The sample with the anomalous copper concentration (at 250 °C and 3.5 Pa) can be considered as an exception. Despite its high absorption in the entire spectrum, an apparently direct gap of only 1.94 eV can still be detected.

Table 6 also shows the Urbach energy values *E*_U_ obtained from the fits. The transition from Tauc-indirect absorption to Urbach exponential absorption is also supposed to be smooth for a certain transition energy *E*_1_; the fitting model assumes that both *α*(*E*) and the derivative d*α*/d*E* vary continuously for *E*_1_ [41]. The values of energy *E*_1_ are presented in Table 6, along with the values of *E*_2_. The *E*_U_ values obtained were above 200 meV, which would indicate a high structural disorder of the material. The samples deposited at 3.5 Pa exhibited the highest *E*_U_ values, exceeding 300 meV. It appears evident that increasing the working pressure to 5.0 improves the structural quality of the material. Even in this case, a second absorption front was observed in the samples deposited at substrate temperatures above 200 °C. The indirect Urbach–Tauc model, with a new Urbach energy of approximately 140 meV, can also fit it. Figure 8B shows the experimental data corresponding to the sample deposited at 200 °C with the theoretical fit of the two absorption fronts.

For a photovoltaic application, it is essential to consider two key parameters: the direct bandgap and the absorption coefficient at 1 eV. These parameters determine the efficiency of a material in converting solar energy into electricity. Here are the most suitable values for a photovoltaic application based on the provided data; in the case of the Cu_3_N thin films deposited at a pressure of 3.5 Pa, it was observed that the direct bandgap at 100 °C was relatively low, with a value of 2.33 eV. This is beneficial as a lower bandgap allows for better absorption of photons from sunlight. Furthermore, at 150 °C, the absorption coefficient at 1 eV was high (7.1 × 10^3^ cm⁻^1^), suggesting that the material has an effective capacity to capture sunlight at this energy. Considering these factors, an intermediate temperature or a combination of both (100 °C and 150 °C) could be suitable for a photovoltaic application, depending on other factors, such as material stability and availability under practical conditions. In the case of Cu_3_N thin films deposited at a pressure of 5.0 Pa, the lowest values of the direct bandgap were found at 100 °C (2.33 eV) and 150 °C (2.14 eV). These values are favourable for the efficient absorption of photons from sunlight in a photovoltaic application, as they indicate that less energy is required to excite electrons. However, it is also crucial to consider the absorption coefficient at 1 eV. At 100 °C, this coefficient is particularly high (7.1 ×·10^3^ cm⁻^1^), implying excellent material capability to capture sunlight at that specific energy. In summary, the data suggest that the temperature of 100 °C is especially promising for photovoltaic applications due to the combination of a relatively low direct bandgap and a high absorption coefficient at 1 eV. 

## 4. Conclusions

This study focused on the fabrication of Cu_3_N thin films by reactive RF magnetron sputtering by using pure nitrogen as the deposition gas. Silicon and glass substrates were employed, and the temperature and pressure conditions varied during the deposition process. The results obtained demonstrated a close relationship between the Cu_3_N properties and the processing conditions.

At temperatures higher than 200 °C, a significant impact was observed in the film properties due to the metastable nature of Cu_3_N. This led to changes in their structural, chemical, mechanical, and optical characteristics. In particular, an increase in grain size was observed as the deposition temperature rose. In addition, a variation in the Cu/N ratio was detected, suggesting a potential improvement in chemical stability at lower substrate temperatures. SEM images revealed flat surfaces with a typical granular morphology. This makes the material sputtered at low temperatures highly suitable for use as a solar absorber. The results underline the importance of knowing the effect of such sputtering parameters to tailor the morphological and structural properties of the Cu_3_N thin films. Finally, optical properties showed that the Cu_3_N thin films deposited at substrate temperatures up to 150 °C exhibited optimal energy bands for solar photon absorption. This renders these materials highly promising for photovoltaic applications. These results underscore the importance of controlling and optimizing the deposition conditions to achieve Cu_3_N thin films with specific properties. Therefore, this study provides a detailed insight into how the deposition conditions can strongly influence the properties of Cu_3_N thin films, which could drive significant advances in the application of nanomaterials in emerging technologies.

## Figures and Tables

**Table 1:** Film thickness and deposition rate obtained for the Cu_3_N thin films deposited at different substrate temperatures and working pressures, deposited on a Corning glass substrate.

**Working Pressure: 3.5 Pa**					
Substrate temperature (°C)	RT	100	150	200	250
Film thickness (nm)	91 ± 6	132 ± 20	128 ± 18	110 ± 15	85 ± 3
Deposition rate (nm/min)	3.03	4.40	4.27	3.67	2.83
**Working Pressure: 5.0 Pa**					
Substrate temperature (°C)	RT	100	150	200	250
Film thickness (nm)	85 ± 5	132 ± 21	119 ± 16	118 ± 15	88 ± 5
Deposition rate (nm/min)	2.83	4.40	3.97	3.93	2.93

**Table 2:** Structural parameters obtained for Cu_3_N thin films deposited at different temperatures and two working pressures.

**Working Pressure: 3.5 Pa**							
T substrate (°C)	Preferred orientation	FWHM (°)	Lattice constant a (nm)	τ (nm)	ε	δ (lines/nm^2^)	N_c_ (nm^−2^)
RT	(100)	0.2460	0.3826	33	5.22 × 10^−3^	9.20 × 10^−4^	1.07 × 10^−5^
100	(100)	0.1573	0.3820	52	3.33 × 10^−3^	3.76 × 10^−4^	2.79 × 10^−6^
150	(100)	0.1378	0.3822	59	2.92 × 10^−3^	2.89 × 10^−4^	1.87 × 10^−6^
200	(100)	0.1181	0.3815	69	2.50 × 10^−3^	2.12 × 10^−4^	1.18 × 10^−6^
250	(111)	0.0590	0.3517	144	6.89 × 10^−4^	4.84 × 10^−5^	6.84 × 10^−8^
**Working Pressure: 5.0 Pa**							
T substrate (°C)	Preferred orientation	FWHM (°)	Lattice constant a (nm)	τ (nm)	ε	δ (lines/nm^2^)	Nc (nm^−2^)
RT	(100)	0.2263	0.3832	36	4.81 × 10^−3^	7.79 × 10^−4^	8.32 × 10^−6^
100	(100)	0.1476	0.3810	55	3.12 × 10^−3^	3.31 × 10^−4^	2.30 × 10^−6^
150	(100)	0.1279	0.3838	63	2.72 × 10^−3^	2.49 × 10^−4^	1.51 × 10^−6^
200	(100)	0.1175	0.3815	69	2.48 × 10^−3^	2.10 × 10^−4^	1.16 × 10^−6^
250	(100)	0.0590	0.3823	137	1.25 × 10^−3^	5.29 × 10^−5^	1.47 × 10^−7^

**Table 3:** Values of atomic concentration (at. %) of Cu_3_N thin films, determined by RBS, at different temperatures for the two working pressures, deposited on the Silicon substrate.

**Working Pressure 3.5 Pa**					
T substrate (°C)	Cu%	N%	O%	C%	Ratio Cu/N
RT	73.5	26.5	-	-	2.8
100	74.1	25.9	-	-	2.9
150	73.3	26.7	-	-	2.7
200	70	30	-	-	2.3
250	76	12	12	-	6.3
**Working Pressure 5.0 Pa**					
T substrate (°C)	Cu%	N%	O%	C%	Ratio Cu/N
RT	71.3	28.7	-	-	2.5
100	72.1	27.9	-	-	2.6
150	73.4	26.6	-	-	2.8
200	62.4	24	13.6	-	2.6
250	67	21.5	1.0	10.5	3.6

**Table 4:** Relationship between Raman shift and microstrain obtained for the Cu_3_N films deposited on silicon at different substrate temperatures.

**Working Pressure 3.5 Pa**		
T substrate (°C)	Raman Shift (cm^−1^)	Microstrain
RT	628 ± 3.4	5.22 × 10^−3^
100	632 ± 0.6	3.33 × 10^−3^
150	632 ± 0.6	2.92 × 10^−3^
200	633 ± 1.6	2.50 × 10^−3^
250	632 ± 0.6	6.89 × 10^−4^
**Working Pressure 5.0 Pa**		
T substrate (°C)	Raman Shift (cm^−1^)	Microstrain
RT	628 ± 4.2	4.81 × 10^−3^
100	627 ± 5.2	3.12 × 10^−3^
150	632 ± 0.2	2.72 × 10^−3^
200	637 ± 4.8	2.48 × 10^−3^
250	637 ± 4.8	1.25 × 10^−3^

**Table 5:** Elastic modulus and hardness of Cu_3_N films at different temperatures and pressures.

**Working Pressure 3.5 Pa**		
T substrate (°C)	Elastic modulus (GPa)	Hardness (GPa)
RT	85.92 ± 4.74	4.55 ± 0.21
100	90.44 ± 5.11	4.47 ± 0.22
150	99.85 ± 9.86	4.36 ± 0.50
200	89.10 ± 3.87	3.81 ± 0.15
250	80.49 ± 7.87	1.40 ± 0.15
**Working Pressure 5.0 Pa**		
T substrate (°C)	Elastic modulus (GPa)	Hardness (GPa)
RT	78.66 ± 3.94	3.86 ± 0.21
100	85.97 ± 6.10	3.58 ± 0.21
150	82.19 ± 3.80	4.30 ± 0.19
200	80.43 ± 2.77	3.17 ± 0.19
250	75.46 ± 2.43	2.78 ± 0.09

**Table 6:** Values of the optical properties of the Cu_3_N layers deposited at different temperatures and working pressures of 3.5 and 5.0 Pa.

**Working Pressure 3.5 Pa**						
T substrate (°C)	Refractive index	Urbach energy (meV)	Indirect bandgap (eV)	Direct bandgap (eV)	Transition energies (eV)	Absorption coeff. at 1 eV (cm^−1^)
RT	2.43	305	1.66	2.29	2.20/2.50	1.1 × 10^3^
100	2.59	246	1.46	2.13	1.90/2.35	6.6 × 10^2^
150	2.54	343	1.62	2.26	2.22/2.47	9.1 × 10^3^
200	2.33	316	1.81	2.38	2.37/2.57	2.2 × 10^3^
250	-	-	-	1.94	-	1.6 × 10^5^
**Working Pressure 5.0 Pa**						
T substrate (°C)	Refractive index	Urbach energy (meV)	Indirect bandgap (eV)	Direct bandgap (eV)	Transition energies (eV)	Absorption coeff. at 1 eV (cm^−1^)
RT	2.59	211	1.50	2.10	1.88/2.30	1.2 × 10^3^
100	2.34	285	1.76	2.33	2.26/2.52	7.1 × 10^3^
150	2.48	268	1.49	2.14	1.97/2.36	1.2 × 10^3^
200	2.26	263 (139)	1.77 (1.37)	2.37	2.24/2.56	2.6 × 10^2^
250	2.15	215 (143)	1.80 (1.38)	2.41	2.20/2.62	1.2 × 10^2^

## Data Availability

Data are available upon request.
